# Genome-Wide Association Mapping and Identification of Candidate Genes for the Rumpless and Ear-tufted Traits of the Araucana Chicken

**DOI:** 10.1371/journal.pone.0040974

**Published:** 2012-07-23

**Authors:** Rooksana E. Noorai, Nowlan H. Freese, Lindsay M. Wright, Susan C. Chapman, Leigh Anne Clark

**Affiliations:** 1 Department of Genetics and Biochemistry, Clemson University, Clemson, South Carolina, United States of America; 2 Department of Biological Sciences, Clemson University, Clemson, South Carolina, United States of America; Universitat Pompeu Fabra, Spain

## Abstract

Araucana chickens are known for their rounded, tailless rumps and tufted ears. Inheritance studies have shown that the rumpless (*Rp*) and ear-tufted (*Et*) loci each act in an autosomal dominant fashion, segregate independently, and are associated with an increased rate of embryonic mortality. To find genomic regions associated with *Rp* and *Et*, we generated genome-wide SNP profiles for a diverse population of 60 Araucana chickens using the 60 K chicken SNP BeadChip. Genome-wide association studies using 40 rumpless and 11 tailed birds showed a strong association with rumpless on Gga 2 (*P*_raw_ = 2.45×10^−10^, *P*_genome_ = 0.00575), and analysis of genotypes revealed a 2.14 Mb haplotype shared by all rumpless birds. Within this haplotype, a 0.74 Mb critical interval containing two *Iroquois* homeobox genes, *Irx1* and *Irx2*, was unique to rumpless Araucana chickens. *Irx1* and *Irx2* are central for developmental prepatterning, but neither gene is known to have a role in mechanisms leading to caudal development. A second genome-wide association analysis using 30 ear-tufted and 28 non-tufted birds revealed an association with tufted on Gga 15 (*P*_raw_ = 6.61×10^−7^, *P*_genome_ = 0.0981). We identified a 0.58 Mb haplotype common to tufted birds and harboring 7 genes. Because homozygosity for *Et* is nearly 100% lethal, we employed a heterozygosity mapping approach to prioritize candidate gene selection. A 60 kb region heterozygous in all Araucana chickens contains the complete coding sequence for *TBX1* and partial sequence for *GNB1L*. *TBX1* is an important transcriptional regulator of embryonic development and a key genetic determinant of human DiGeorge syndrome. Herein, we describe localization of *Rp* and *Et* and identification of positional candidate genes.

## Introduction

There are hundreds of domestic chicken breeds worldwide [1]. Breeds were generally developed for meat and egg production, but morphological traits, plumage color, and other distinctive characteristics were also selected. The Araucana chicken, originally from Chile, is a multi-purpose breed initially established for its blue-shelled eggs [1], [2]. Araucana chickens are also known for two other distinguishing traits: a rounded, tailless rump and protruding ear-tufts. Although these traits segregate in the population, the United States Araucana breed standard requires show birds to possess both phenotypes.

The rumpless phenotype is characterized by the absence of all free caudal vertebrae and the uropygial gland [3]. Without underlying skeletal support, birds with caudal truncation lack a fleshy rump and tail feathers [3]. An intermediate rumpless phenotype, wherein some caudal vertebrae are present but irregularly fused together, is thought to result from a modifier gene introduced through crosses with non-Araucana tailed chickens [3], [4]. The rumpless phenotype arises from a defect in caudal patterning that is controlled by a dominant gene (*Rp*) [3]. Rumpless Araucana chickens may be heterozygous or homozygous for this locus. In test matings, all rumpless intermediates were determined to be heterozygous (*Rp*/*rp* ^+^) [3]. Homozygosity is underrepresented among chicks from rumpless to rumpless matings, indicating that the *Rp*/*Rp* genotype has reduced viability [3], [5]. Birds having at least one copy of *Rp* have increased mortality in the embryo stage, with death occurring at 17 to 21 days of incubation [3]. Rumpless birds also have reduced fecundity as adults [3].

**Figure 1:**
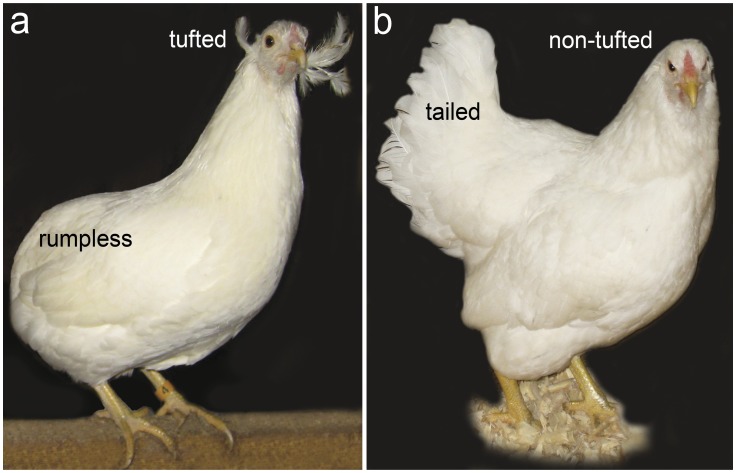
Araucana chicken. (**a**) General appearance of a rumpless, tufted Araucana chicken. (**b**) For comparison, a tailed, non-tufted Araucana chicken.

Ear-tufts are feather-covered, epidermal protrusions originating near the ear canal (Figure 1). The mass of tissue forming the protrusion, or peduncle, is believed to develop as a result of the incomplete fusion of the hyomandibular arches, and it can vary in position and length (from 2 mm to 2 cm) [6], [7]. Tufted chickens may also have structural rearrangement of the ears [6]. Abnormalities include irregularly shaped external ear openings and shortened or absent external auditory canals [6].

Inheritance studies indicate that tufted is governed by a dominant locus, *Et* [6], [8]. Test matings show that all tufted birds are heterozygous (*Et/et* ^+^) and that homozygosit*y* for *Et* is lethal at about 17–19 days of incubation [6], [8]. Lethality among a portion of heterozygous birds is also reported, appearing to occur at 20–21 days of incubation [8]. Post-hatch mortality is significantly higher among tufted chickens [6], [8].

Because tufts can occur unilaterally or bilaterally and may differ in size from one side to the other, *Et* is proposed to have variable expressivity [6]. In addition, a paucity of tufted progeny from mating studies in 1978 suggests reduced penetrance of the tufted locus [6]. In 1981, Somes and Pabilonia identified a tufted male that produced excessive tufted progeny when crossed with an *et* ^+^/*et* ^+^ White Leghorn (86%), and they speculated that *Et*/*Et* birds may occasionally reach maturity [8]. The non-tufted chicks from the *Et*/*Et* male produced tufted progeny when crossed with an *et* ^+^/*et* ^+^ White Leghorn, indicating that their predicted genotype does not match their phenotype, providing further evidence for variable penetrance.

The aim of our investigation was to localize the genetic bases for the rumpless and tufted phenotypes of the Araucana chicken. To this end, we generated genome-wide SNP profiles for 60 Araucana chickens using the 60 K chicken SNP BeadChip [9]. Using a genome-wide association approach, we elucidate the chromosomal regions harboring *Rp* and *Et* and identify strong candidate genes for each trait.

## Results

**Figure 2:**
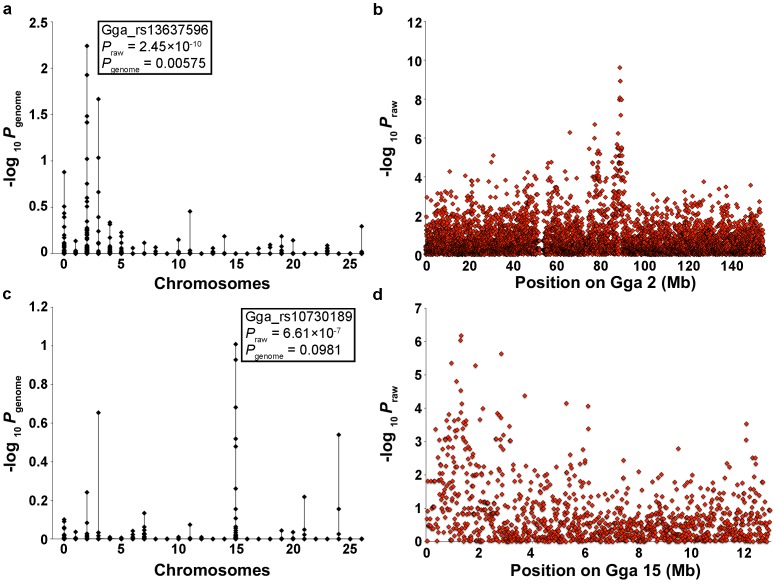
Genome-wide association for *Rp* and *Et*. After 100,000 permutations, the genome-wide adjusted *P* values (−log_10_ *P*_genome_) for each SNP are plotted by chromosome (left). The raw *P* values for the most strongly associated chromosomes are plotted against chromosomal position (right). (**a,b**) 40 rumpless versus 11 tailed Araucana chickens (**c,d**) 30 tufted versus 28 non-tufted Araucana chickens.

Case/control analyses were carried out using 40 rumpless and 11 tailed Araucana chickens (Figure 2a). Seven birds described as having partial tails by their breeders were excluded from the rumpless association analysis because of uncertainty concerning their phenotype. A total of 191 SNPs were associated with the rumpless phenotype (*P*_raw_ ≤0.0001), 72 of which were located on Gga 2 (Figure 2b). The most significant result obtained was for SNP Gga_rs13637596, located on chromosome 2 at position 88.95 Mb (*P*_raw_ = 2.45×10^−10^, *P*_genome_ = 0.00575). The next two most significant results were for proximal SNPs located at 89.17 Mb (*P*_raw_ = 1.20×10^−9^, *P*_genome_ = 0.0119) and 89.19 Mb (*P*_raw_ = 1.20×10^−9^, *P*_genome_ = 0.0119).

**Figure 3:**
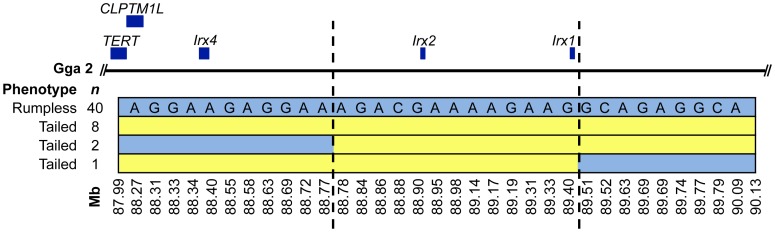
Localization of *Rp*. Physical map showing the relative positions of mapped genes and informative SNP markers within the 2.14 Mb rumpless haplotype on Gga 2. Light blue shading denotes the rumpless haplotype (alleles are shown in the top row). Dashed lines flank the critical interval wherein no tailed birds share the rumpless haplotype.

Analysis of genotypes in the Gga 2 region revealed a 2.14 Mb haplotype (87.99–90.13 Mb) predicted to contain five genes (Figure 3). All 40 rumpless birds had at least one copy of the haplotype: 18 were homozygous and 22 were heterozygous. Partial tailed birds were heterozygous. The haplotype was absent in its entirety from the 11 tailed birds. Three tailed birds were heterozygous for partial blocks of the haplotype and further delimit the critical interval to 0.74 Mb (88.77–89.51 Mb). This region contains two candidate genes: *Irx1* and *Irx2*.

Analyses for association with the tufted phenotype, using 30 cases and 28 controls, resulted in 31 significant SNPs, 11 of which map to Gga 15 (Figure 2c). The most significant results were for SNPs Gga_rs10730189 (*P*_raw_ = 6.61×10^−7^, *P*_genome_ = 0.0981) and Gga_rs15762547 (*P*_raw_ = 9.19×10^−7^, *P*_genome_ = 0.118), located at positions 1.33 Mb and 1.30 Mb on chromosome 15, respectively. Four other proximal SNPs also reached significance (Figure 2d).

**Figure 4:**
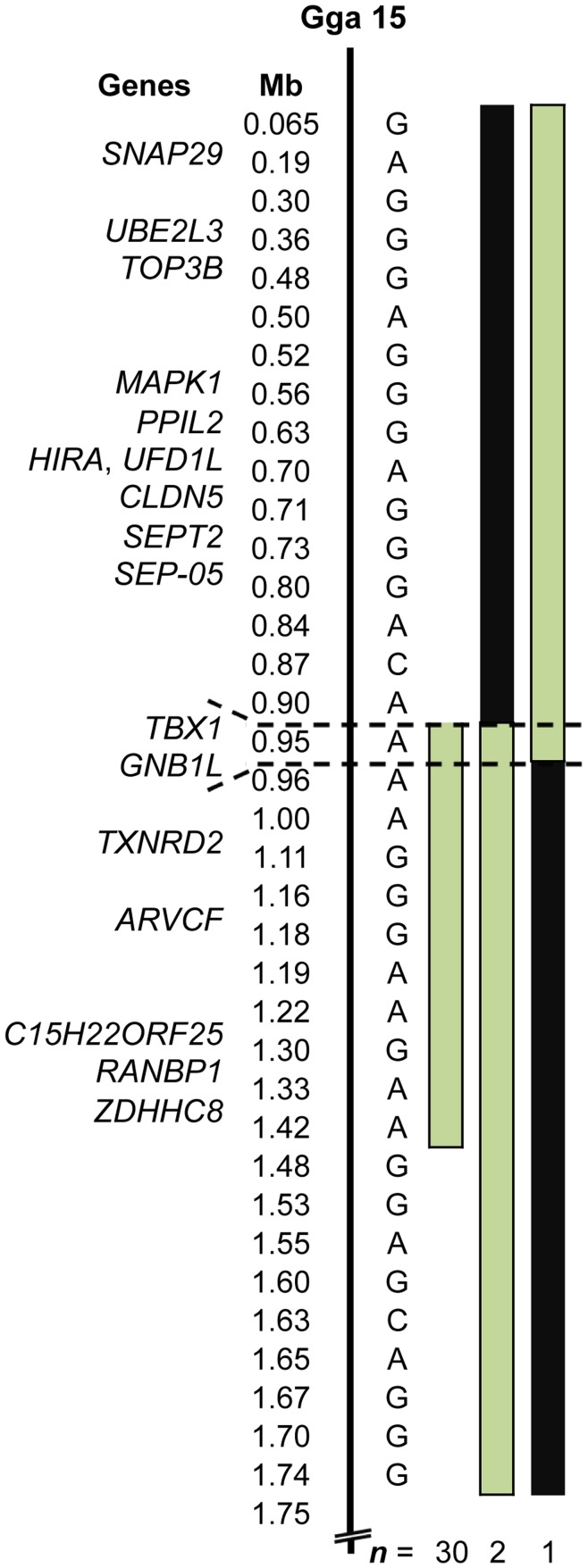
Localization of *Et*. Physical map showing the relative positions of genes and informative SNP markers in the associated region of Gga 15. Alleles of the tufted haplotype and positions are shown. Pale green bars denote heterozygosity for the tufted haplotype. Black bars denote homozygosity for the tufted haplotype. Dashed lines mark a 60 kb interval wherein all tufted birds are heterozygous for the haplotype.

Analysis of genotypes reveals that 29 of 30 tufted birds shared a haplotype extending from the telomere of Gga 15 to position 1.75 Mb. These birds were heterozygous for the complete haplotype. Two of 28 non-tufted birds were also heterozygous for the haplotype in its entirety. A single tufted bird shared only part of the 1.75 Mb haplotype, defining a 0.58 Mb (0.90–1.48 Mb) critical interval that is heterozygous in all 30 tufted birds and contains 7 genes. Because tufted is nearly always recessive lethal, blocks of homozygosity for the tufted haplotype were identified to reduce the number of candidate genes. Homozygosity blocks in three birds flank a 60 kb interval harboring two genes: *TBX1* and *GNB1L* (Figure 4).

## Discussion

In this study, we used genome-wide SNP profiles to localize genes causative for two breed-defining phenotypes of Araucana chickens, rumpless and ear-tufts. We took advantage of the fact that both traits segregate independently in the population by using a single data set to carry out an association analysis for each trait. Haplotype analyses based on inheritance patterns were used to identify positional candidate genes for both traits.

We identified a rumpless haplotype spanning 2.14 Mb and five genes on chromosome 2. The haplotype is present in the heterozygous or homozygous state in rumpless birds. All 7 birds with partial tails are heterozygous for the rumpless haplotype and likely represent the intermediate phenotype described by Dunn and Landauer [3]. Because rumpless is dominant and fully penetrant, we further delimited the critical interval by identifying regions of the haplotype shared by tailed birds. A 0.74 Mb region common to all rumpless birds, and absent from 11 tailed birds, harbors *Rp*.

These data reveal that *Rp* maps to a region of Gga 2 that is distinct from the predicted location of genes previously associated with caudal truncation [10]–[14]. The 0.74 Mb critical interval contains the *Iroquois* homeobox genes, *Irx1* and *Irx2.* The *Iroquois* genes encode transcription factors that function in patterning and regionalization of tissues early in development [15]. *Irx1* and *Irx2* are prepattern and proneural genes first identified in *Drosophila* and *Xenopus* [16], [17]. Studies of gene function suggest that *Irx* genes have redundant yet distinct roles in development [18], [19]. *Irx* genes have been knocked out in mice and zebrafish with little effect on tail development [19]–[23]. However, the rumpless phenotype is dominant, suggesting that misexpression of *Irx1* or *Irx2* may underlie the trait, rather than loss of function.

We identified SNPs on Gga 15 that are strongly associated with the tufted phenotype and define a 0.58 Mb haplotype for which all tufted birds in our cohort are heterozygous. No birds are homozygous for the complete tufted haplotype. These data support conclusions from previous inheritance studies that suggest nearly 100% of tufted birds are heterozygous, and that *Et*/*Et* is lethal [6], [8].

Two non-tufted Araucana chickens are heterozygous for the tufted haplotype. These birds may signify reduced penetrance. Penetrance of the tufted allele is estimated to range from 86% to 96% [6], [8]. Based on the assigned phenotypes and the associated haplotype, we observed 94% penetrance in our cohort. Alternatively, these birds may have been incorrectly phenotyped by their breeders due to short peduncles or missing protruding feathers.

The 0.58 Mb haplotype harbors 7 protein-coding genes. Unlike rumpless, identification of the tufted haplotype in non-tufted birds could not be used to narrow the critical interval because of reduced penetrance. However, because homozygosity for *Et* is nearly always lethal, we were able to prioritize candidate gene selection using heterozygosity mapping. Tufted birds with blocks of homozygosity extending into the 0.58 Mb common haplotype were identified, and these regions were deemed less likely to harbor the *Et* locus. These data indicate that *Et* is located in a region containing partial coding sequence for *GNB1L*, which encodes a protein implicated in neuropsychiatric disorders [24], [25], and complete coding sequence for *TBX1* [26], an important transcriptional regulator of embryonic development.

Haploinsufficiency for *TBX1* is considered to be the key genetic determinant of human DiGeorge syndrome (DGS), which is caused by a heterozygous chromosomal deletion of 22q11.2 [27]. While the clinical phenotype is highly variable, DGS is characterized by craniofacial and cardiovascular abnormalities. Malformations in DGS are attributed to disturbed segmentation and patterning of the pharyngeal structures [28]. Auricular defects common in DGS include narrow or absent external ear canal and protruding ears [29]. Homozygosity for null mutations of *TBX1* in mice and zebrafish causes a range of phenotypic effects similar to DGS, including abnormal ear development [30], [31]. Based on phenotypic similarities between the malformations causing ear tufts and DGS, *TBX1* is a highly plausible candidate gene and the primary focus of ongoing work to identify the genetic basis for ear-tufts in Araucana chickens.

In conclusion, we used genome-wide association and haplotype analyses to localize *Rp* and *Et* to chicken chromosomes 2 and 15, respectively. In addition, we identified candidate genes that are immediate targets for future work.

## Materials and Methods

### Ethics Statement

This study was approved by the Clemson University IACUC protocol number 2011-041 and IBC protocol number 2010-041.

### Study Cohort

Whole blood for DNA was collected from 6 different flocks of Araucana chickens from the United States. Phenotypic information and photographs, when available, were provided by owners. Birds with tufts of any size and on either side of the head were classified as tufted. Because both traits segregate in the Araucana population, birds were selected to ensure that the phenotypes were balanced. Our study cohort comprised 60 Araucana chickens: 21 rumpless/tufted birds, 20 rumpless/non-tufted birds, 7 tailed/non-tufted birds, 5 tailed/tufted birds, 5 partial/tufted birds, and 2 partial/non-tufted birds. Genomic DNA was isolated using the DNeasy blood and tissue kit (QIAGEN, Valencia, USA) and adjusted to a concentration of 50 ng/uL.

### Genome-wide Association Mapping

SNP genotypes were generated using the Illumina 60 K chicken SNP BeadChip, which has 57,636 SNPs across chromosomes 1 through 28, Z, W, and two unmapped linkage groups [9]. BeadChips were processed by DNA Landmarks (Quebec, Canada), according to manufacturer’s protocols. Raw data files were analyzed using GenomeStudio’s Genotyping Module to generate SNP calls. The PLINK Input Report Plug-in v2.1.1 was used to format the data. For analysis, Gga 27, Gga 28, Gga Z, Gga W, and microchromosomes were all identified as chromosome zero. Case/control analyses using 56,685 SNPs were performed using PLINK [32]. Two birds with excessive missing data were excluded from all analyses. By convention, *P*_raw_ values ≤0.0001 were considered significant. Permutation testing, using 100,000 iterations, was carried out using PLINK.
